# Effect of classroom-based physical activity interventions on academic and physical activity outcomes: a systematic review and meta-analysis

**DOI:** 10.1186/s12966-017-0569-9

**Published:** 2017-08-25

**Authors:** Amanda Watson, Anna Timperio, Helen Brown, Keren Best, Kylie D. Hesketh

**Affiliations:** Institute for Physical Activity and Nutrition (IPAN), School of Exercise and Nutrition Science, Deakin University, Geelong, Australia

**Keywords:** Classroom, Physical activity, Academic performance, Children, Schools, Intervention, Systematic review, Meta-analysis

## Abstract

**Background:**

Physical activity is associated with many physical and mental health benefits, however many children do not meet the national physical activity guidelines. While schools provide an ideal setting to promote children’s physical activity, adding physical activity to the school day can be difficult given time constraints often imposed by competing key learning areas. Classroom-based physical activity may provide an opportunity to increase school-based physical activity while concurrently improving academic-related outcomes. The primary aim of this systematic review and meta-analysis was to evaluate the impact of classroom-based physical activity interventions on academic-related outcomes. A secondary aim was to evaluate the impact of these lessons on physical activity levels over the study duration.

**Methods:**

A systematic search of electronic databases (PubMed, ERIC, SPORTDiscus, PsycINFO) was performed in January 2016 and updated in January 2017. Studies that investigated the association between classroom-based physical activity interventions and academic-related outcomes in primary (elementary) school-aged children were included. Meta-analyses were conducted in Review Manager, with effect sizes calculated separately for each outcome assessed.

**Results:**

Thirty-nine articles met the inclusion criteria for the review, and 16 provided sufficient data and appropriate design for inclusion in the meta-analyses. Studies investigated a range of academic-related outcomes including classroom behaviour (e.g. on-task behaviour), cognitive functions (e.g. executive function), and academic achievement (e.g. standardised test scores). Results of the meta-analyses showed classroom-based physical activity had a positive effect on improving on-task and reducing off-task classroom behaviour (standardised mean difference = 0.60 (95% CI: 0.20,1.00)), and led to improvements in academic achievement when a progress monitoring tool was used (standardised mean difference = 1.03 (95% CI: 0.22,1.84)). However, no effect was found for cognitive functions (standardised mean difference = 0.33 (95% CI: -0.11,0.77)) or physical activity (standardised mean difference = 0.40 (95% CI: -1.15,0.95)).

**Conclusions:**

Results suggest classroom-based physical activity may have a positive impact on academic-related outcomes. However, it is not possible to draw definitive conclusions due to the level of heterogeneity in intervention components and academic-related outcomes assessed. Future studies should consider the intervention period when selecting academic-related outcome measures, and use an objective measure of physical activity to determine intervention fidelity and effects on overall physical activity levels.

## Background

Multiple physical and mental health benefits can be attained when children participate in the recommended 60 min per day of moderate- to vigorous-intensity physical activity [1, 2]. Despite these benefits, population based-studies have reported that over 50% of children in Australia and internationally are not meeting recommendations [3–6]. Schools are considered ideal settings for the promotion of children’s physical activity. There are multiple opportunities for children to be physically active over the course of the school week, including during break times, sport, Physical Education class and active travel to and from school. Studies have shown interventions targeting these discrete periods may be effective in increasing children’s physical activity levels [7, 8], with the potential to contribute to up to 50% of the physical activity required to meet physical activity guidelines [9]. However, with limited time available during these discrete periods, additional opportunities may be required in order for children to achieve the recommended levels of physical activity. Classroom-based physical activity provides another way for children to be active at school. This involves classroom teachers incorporating physical activity into class time through either integrating physical activity into lessons (physically active lessons), or adding short bursts of physical activity, either with curriculum content (curriculum focused active breaks) or without (active breaks).

There is increasing interest from researchers and education professionals about the potential for classroom-based physical activity to positively impact academic-related outcomes, including classroom behaviour, cognitive function and academic achievement. While some teachers express concern that classroom-based physical activity may have an adverse effect on on-task classroom behaviour [10], emerging evidence from systematic reviews and meta-analyses suggest that overall physical activity may have a small positive effect on on-task classroom behaviour [11–17]. There is less evidence on classroom-based physical activity.

Narrative reviews [18–20], one systematic review [21] and two meta-analyses [22, 23] have explored the impact of classroom-based physical activity interventions on academic-related outcomes. However, these were narrow in scope, included few studies, and combined findings among primary and secondary school students, which may be problematic due to the difference in education settings.

A systematic review of 11 studies concluded that physically active lessons may have a positive effect, or no effect on academic-related outcomes [21]. However, that study did not consider other forms of classroom-based physical activity (e.g. active breaks), combined findings among primary and secondary school students, and did not include a meta-analysis [21].

A meta-analysis of four intervention studies found that classroom-based physical activity had a positive effect on academic-related outcomes (M = 0.67; 95%CI:0.26,1.09) [23]. Similar results were reported in a meta-analysis of 24 intervention studies investigating the association between different types of physical activity (e.g., during recess or lunch vs. active breaks vs. physically active lessons) and school engagement (behaviour at home and at school, and emotions, e.g. lesson enjoyment) [22]. In that meta-analysis, overall results showed physical activity had a significant positive effect on school engagement (d = 0.28;95%CI:0.12,0.46) [22]. When broken down into type of physical activity, active breaks (*n* = 4 studies) appeared to be the most effective type of intervention for improving school engagement (d = 0.55; 95%CI:0.02,1.06), compared with recess or lunch time physical activity (*n* = 3 studies; d = 0.26; 95%CI:-0.19,0.73) and physically active lessons (*n* = 5 studies; d = 0.22; 95%CI: -0.21,0.66) [22]. However, results from those meta-analyses are limited by the small number of included studies [22, 23], the narrow range of potential academic-related outcomes assessed, the combination of findings among primary and secondary school students [22], and their recency [23].

The current paper aims to expand on findings from these reviews by conducting a systematic review and meta-analyses of the evidence of effect of classroom-based physical activity interventions (active breaks, curriculum-focused active breaks and physically active lessons) on a broad range of academic-related outcomes (classroom behavior, cognitive function and academic achievement), specifically among primary school-aged children. A secondary aim is to examine the effect of these interventions on children’s physical activity levels.

## Methods

### Definitions

While there are no set definitions for classroom-based physical activity, the following definitions are provided in order to maintain consistency and clarity throughout the remainder of this systematic review.

*Classroom-based physical activity:* physical activity carried out during regular class time, and can occur either inside or outside the classroom (e.g. hallway, playground), and is distinct from school recess/lunch break times. Classroom-based physical activity can take three forms:

- *Active breaks:* short bouts of physical activity performed as a break from academic instruction [24].
- *Curriculum-focussed active breaks:* short bouts of physical activity that include curriculum content [25, 26].
- *Physically active lessons*: the integration of physical activity into lessons in key learning areas other than physical education (e.g. mathematics) [27, 28].

*Academic-related outcomes:* overarching term to encompass factors associated with academic performance at school. These can be grouped into three main categories:

- *Classroom behaviour:* Observed behaviours that may promote or interfere with learning in the classroom, including on-task behaviour [29] (e.g. concentrating on tasks assigned by the teacher), and off-task behaviour (e.g. not concentrating on tasks assigned by the teacher).
- *Cognitive function:* Mental process (e.g. executive function) that may influence academic performance [29].
- *Academic achievement:* A child’s performance on school-related tasks; often reported via classroom grades, national standardised tests or progress monitoring tools [29], as well as self-reported perceived academic competence [30].

### Registration and protocol

This study followed the Preferred Reporting Items for Systematic Reviews and Meta-Analyses (PRISMA) recommendations for systematic review reporting, and was registered with the International Prospective Register of Systematic Reviews (PROSPERO) (record #CRD42016027294).

### Search strategy

Studies were identified through a systematic search of four electronic databases (PubMed, ERIC, SPORTDiscus and PsycINFO), first conducted in January 2016, and updated in January 2017 by one author (AW). The search strategy consisted of four elements (see Table 1). The search was limited to peer-reviewed articles published in English in all available years. ‘Grey’ literature, including the reference lists from the websites of two organisations (“Active Academics” and “Active Living Research”) involved in children’s physical activity research were also searched.

**Table 1:** Article search terms and databases searched

Classroom-based	Physical activity	Academic-related outcomes	Study population	Database searched
Classroom[tiab] OR break*[tiab] OR curricul*[tiab] OR “active break”[tiab] OR integrat*[tiab] OR lesson*[tiab]	“Physical activity”[tiab] OR “physically active”[tiab] OR exercis*[tiab] OR active[tiab] OR activity[tiab]	Educational status[tiab] OR educational measurement [mh:noexp] OR cognition[mh:noexp] OR Academic[tiab] OR “Grade point average”[tiab] OR “Standardised test scores”[tiab] OR “standardized test scores”[tiab] OR “test scores”[tiab] OR Reading[tiab] OR Math*[tiab] OR learn*[tiab] OR grade*[tiab] OR literacy[tiab] OR numeracy[tiab] OR academic[tiab] OR attent*[tiab] OR Concentration[tiab] OR behaviour[tiab] OR behavior[tiab] OR cogniti*[tiab] OR “executive function”[tiab] OR “fluid intelligence”[tiab] OR achievement[tiab] OR learning[tiab]	Student[tiab] OR Student*[tiab] OR child[mh] OR child*[tiab] OR class*[tiab]	PubMed
classroom or school or lesson	physical activity or exercise	academic or achievement or cognitive	children or child or student or class	SPORTDiscus ERIC PsycINFO

### Inclusion criteria

A predetermined set of inclusion criteria were used to select papers for this systematic review. Each study had to meet the following criteria:

1. Intervention study design;
2. Investigated associations between classroom-based physical activity and at least one academic-related outcome. Interventions involving strategies in addition to classroom-based physical activity were excluded (to enable the effects of classroom-based physical activity to be isolated);
3. Study population included primary school-aged children (5–12 years);
4. Presented original data;
5. Did not focus specifically on special populations (e.g. overweight children).

### Study selection

**Fig. 1:**
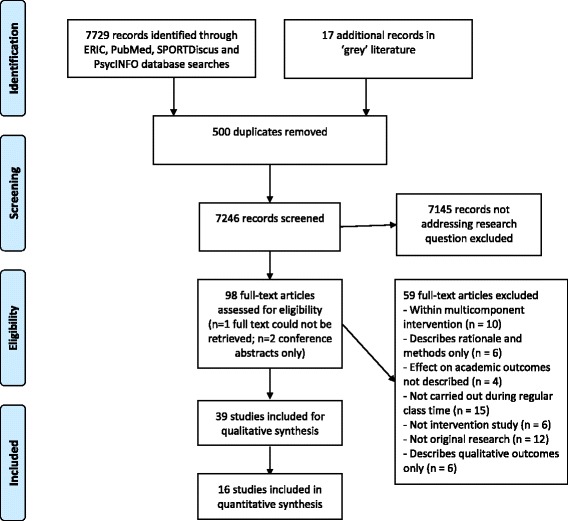
PRISMA Flow Diagram showing flow of studies through the review process

The search yielded 7729 citations from electronic database records, and 17 from ‘grey’ literature (Fig. 1). After removing duplicates (*n* = 500), the titles and/or abstracts of 7246 unique publications were screened by one author (AW). A total of 101 publications were identified as potentially relevant according to the inclusion criteria. Full texts of 98 of these 101 articles were obtained and reviewed independently by two authors to determine eligibility (AW, KB). Two full texts were conference abstracts only, and one full-text was unable to be retrieved despite extensive librarian-assisted enquiries and emails directly to the contact author. Of the 98 full-text articles, a total of 59 were excluded as not meeting inclusion criteria. Disagreements between the two reviewers were resolved through discussion with all authors. Reference lists of included articles were also examined, however no additional studies were identified. Thirty-nine unique citations satisfied the eligibility criteria and were included in this systematic review.

### Data extraction

Paper characteristics including country of study, study design, participant characteristics, intervention characteristics, academic-related outcome measures, physical activity measures, and results were extracted by one author (AW). Interventions were then categorised as active break, curriculum focussed active break, or physically active lesson intervention.

### Methodological quality

Two authors (AW, KB) independently assessed the methodological quality of the included studies using the Effective Public Health Practice Project (EPHPP) tool [31]. This six-component rating scale for interventions assesses (1) selection bias; (2) study design; (3) confounders; (4) blinding; (5) data collection methods; and (6) withdrawals and drop outs. Each component was rated on a three-point scale as either strong, moderate or weak using the tool’s defined criteria. Based on these ratings, an overall methodological quality score was given; either strong (no weak component ratings); moderate (one weak component rating); or weak (more than one weak component rating), following the tool’s accompanying instructions. Where disagreements existed, deliberation occurred until a consensus was reached.

### Meta-analyses

Meta-analyses were conducted where there were at least three studies investigating the same broad outcome, i.e. classroom behaviour, cognitive function, or academic achievement. Due to heterogeneity across study designs, for inclusion studies were required to have a separate comparison group (i.e. RCT or quasi experimental with control group). Studies that used a within subject or cross over study design were therefore excluded from meta-analysis.

To avoid duplication of studies under a single outcome, where studies reported intervention effects on multiple measures for an outcome (this happened only for cognitive functions) [32, 33] a decision was made to include outcomes relating to executive functions, over memory. Executive functions, inhibition in particular, have been shown to be consistently related to academic achievement [34] and therefore were considered salient to teachers. Thus, where inhibition and memory were reported, only inhibition was included in the meta-analysis; where executive functions and short term memory were reported, only executive functions were included in the meta-analysis. Typically higher scores were reflective of better academic-related outcomes. Where lower scores reflected better academic-related outcomes these scores were reversed.

As academic achievement tools varied widely in quality, only studies using national standardised tests or progress monitoring tools were included in the meta-analyses. Further, intervention effects on mathematics were used when studies reported multiple subject assessments, as math was the most commonly reported outcome. Of the 39 studies included in this systematic review, 16 were included in meta-analyses. Reasons for exclusion were: insufficient data for calculating effect sizes and authors did not respond to email requests for additional data (*n* = 6), using a within subject or cross-over study design (*n* = 9), not including a separate comparison group (*n* = 2), insufficient studies investigating an outcome (*n* = 4), or only reporting results separately for subgroups (e.g. BMI categories) (*n* = 2).

## Analysis

Meta-analyses were conducted using Review Manager 5.3. The wide variation in interventions and academic-related outcomes employed in the different studies warranted use of a random effects model. Effect sizes (standardised mean difference) were computed as the difference between treatment and control means.

## Results

Of the 39 studies identified, 19 examined the effect of active breaks [24, 26, 35–51], seven examined curriculum-focussed active breaks [25, 52–57], and thirteen examined physically active lessons [27, 28, 32, 33, 58–66] on academic-related outcomes. The majority of studies (*n* = 27) were published in or after 2014 [24, 26, 32, 33, 36, 39–41, 43, 46–51, 57, 65, 66], and none before 2006. Most (*n* = 18) were conducted in the USA [25, 36, 39, 40, 42, 44, 45, 51–55, 57–60, 64, 65], seven in the Netherlands [32, 41, 49, 50, 61, 62, 66], four in Australia [27, 28, 46, 47], three in Canada [24, 35, 43], two in Scotland [37, 38], and one each in South Africa [48], UK [63], Greece [56] Denmark [33], and Switzerland [26]. Sample sizes ranged from 14 [60] to over 4500 participants [45], with sample sizes <300 in the majority of studies (*n* = 28) [24–28, 33, 35, 39–41, 43, 44, 46–51, 53, 55–57, 59–64]. Intervention periods spanned from single lessons [49, 55, 59, 65] to 3 year duration [58], with most lasting no longer than nine weeks (*n* = 23) [24–28, 33, 37–41, 43–46, 48, 50, 52, 55–57, 59, 63]. Study information is presented in Table 2 (active breaks), Table 3 (curriculum focused active breaks) and Table 4 (physically active lessons).

**Table 2:** Studies investigating the effect of active breaks on physical activity and academic-related outcomes

Paper/ country	Study design	Sample size	Age	Intervention	Duration	Delivery	Physical activity measure	Academic outcome measure	Study quality	Results
*Ma* et al.*, 2014* [34] *Canada*	Within subject	Students: *n* = 44 Schools: *n* = 2 Classes: *n* = 2	Years 2 & 4	FUNtervals = 20s VPA separated by 10s rest repeated 8 times Dose: alternating days	3 weeks	Research staff	None	Off-task behaviour: direct observation	Strong	Off-task behaviour Significantly less following FUNtervals, compared with no activity condition - Year 4 children: off-task passive (ES = 0.31); off-task motor (ES = 0.48) - Year 2 children: off-task passive (ES = 0.74); off-task verbal (ES = 0.45) off-task motor (ES = 1.076)
*Howie* et al.*, 2015* [31] USA	Within subject	*n* = 96	Age 9 to 12 years Years 4 & 5	Brain BITES (Better Ideas Through Exercise) = 5, 10 and 20 min MVPA active breaks Dose: 1 condition delivered twice per week	4 weeks	Research staff	Intervention fidelity: direct observation	Executive function: trail making test & digit recall tests Mathematics: 1-min math fluency test	Moderate	Executive function: no difference between groups Mathematics: significant improvement after 10-min (ES = 0.24) and 20-min (ES = 0.27) active break, compared with sedentary condition
*Howie* et al.*, 2014* [30] *USA*								On-task behaviour: direct observation	Moderate	On-task behaviour: largest improvement after 10 min active break (d = 0.50)
*Janssen* et al.*, 2014* [32] *Netherlands*	Within subject	*n* = 123	Age 10 to 11 years Year 5	15 min active breaks of varying PA intensities (MPA, VPA, passive break, no break) Dose: unclear	4 weeks	Research staff	PA intensity during active breaks: Accelerometer	Selective attention: Test of Everyday Attention for children (TEA-ch test)	Moderate	Selective attention: improved most after MPA condition (*B* = −0.59, 95% CI: −0.70,-0.49), compared with VPA (*B* = −0.29, 95% CI: −0.39,-0.19), passive break (*B* = 0.27, 95% CI: −0.35,-0.18) and no break conditions
*Ma* et al.*, 2015* [21] *Canada*	Within subject	*n* = 88	Age 9 to 11 years Years 3 to 5	FUNtervals = 20s VPA separated by 10s rest, repeated 8 times Dose: once/week	3 weeks	Research staff	None	Selective attention: d2 Test of Attention	Moderate	Selective attention: significant improvement following FUNtervals, compared with no activity condition
*Barnard* et al.*, 2014* [39] *South Africa*	Quasi-experimental with pre and post testing	Students: *n* = 149 Schools: *n* = 2 Classes: *n* = 6	School A mean age: 7.33 years School B mean age: 7.47 years	2 intervention programs: *integrated - 30 min integrated academic skills and motor skill program *intensive program - 30 min physical activity program Dose: 3 times/week	8 weeks	Unclear	None	Literacy: ESSI Reading and Spelling tests Numeracy: VASSI Math Skills Test	Moderate	Reading: for the integrated (26%) and intensive (30%) programs test scores improved but not significant. Spelling: for the integrated (32%) and intensive (47%) programs test scores improved but not significant. Numeracy: for the integrated (30%) and intensive (21%) programs test scores improved but not significant.
*Hill* et al.*, 2011* [29] *Scotland*	Cross over	*n* = 552	Age 8 to 12 years Years 4 to 7	10 to 15 min MPA active break. Dose: once/day for one week, no intervention in the second week	2 weeks	Not reported	None	Attention and executive function: paced serial addition, size ordering, listening \span, digit span backwards & visual coding	Moderate	Attention and executive function: improved only for those receiving the intervention in week 2 (mean difference = 3.85, 95% CI = 0.26,7.44)
*Schmidt* et al.*, 2016* [26]	Within subject	*n* = 98	Year 5	10 min active break involving running at different speeds Dose: 5 different days over 3 weeks	3 weeks	Not reported	None	Attention: d2 Test of Attention	Moderate	Attention: no significant improvement
*Ahamed* et al.*, 2007* [35] *Canada*	Cluster RCT	Students: *n* = 288 Schools: *n* = 10	Age 9 to 11 years Years 4 and 5	Action Schools! BC = 15 min MVPA active break. Dose: once/day	16 months	Teacher	Habitual PA: Modified Physical Activity Questionnaire for Children (PAQ-C)	Mathematics, Reading and Language: Canadian Achievement Test	Weak	Mathematics, Reading and Language (total score) Although control school had significantly higher scores at baseline, no significant difference between intervention (mean = 1672 (9.6) and control groups (mean = 1688.6 (16.6) at follow up Physical activity: increase by 47 min/week in intervention schools (139 ± 62 vs 92 ± 45, *p* < 0.001)
*Carlson* et al.*, 2015* [27] USA	Quasi-experimental (no pre-testing)	Students: *n* = 1322 Teachers>: *n* = 397 Schools: *n* = 24	Mean age: 8.8 years Years 1 to 6	10 min MVPA active break Dose: At least once/day	8 months	Teacher	School day PA: Accelerometer	Classroom behaviour: Teacher report	Weak	Classroom behaviour: Teachers who reported implementing active breaks reported fewer students who lacked effort or gave up easily (β = −0.17, 95% CI: −.033, −0.01), were more likely to agree that students work improves following participation in active breaks (OR = 1.88; 95% CI:1.04,3.37), and showed a trend towards agreement that students stay on task more after active breaks (OR = 1.88; 95% CI: 0.98,3.61; *p* = 0.056), compared with non-implementers Physical activity: students of teachers who reported ever holding active break had 3.14 more minutes per day of MVPA and were 75% more likely to have met the 30 min per day guideline for MVPA during school (OR: 1.75; 95% CI: 1.22, 2.51)
*Hill* et al.*, 2010* [28] *Scotland*	Cross over	*n* = 1224	Age 8 to 12 years Years 4 to 7	10 to 15 min MPA active break. Dose: once/day for one week, no intervention in the second week	2 weeks	Not reported	None	Attention and executive function: paced serial addition, size ordering, listening span, digit span backwards & visual coding	Weak	Attention and executive function: improved only for those receiving the intervention on week 2 of the intervention (control group mean = 58.20 (18.03) vs. intervention group mean = 60.19 (19.38)
*Katz* et al.*, 2010* [33] *USA*	RCT	*n* = 1214	Years 2 to 4	Activity Bursts in the Classroom = MVPA active breaks totaling 30 mins per day. Dose: Length and number of sessions/day could vary	8 months	Teacher	None	Classroom behaviour: Work and social skills component of Independence School District (ISD) progress report card Mathematics and English: Year 4: Missouri Academic Performance Test (MAP) Years 2–4: ISD progress report	Weak	Classroom behaviour: no difference between groups Academic achievement: no difference between groups for MAP test results (Year 4 only), but a greater proportion of control group students (Years 2 to 4) showed improvement in math (28.6% vs. 20.8%) and reading (21.1% vs. 16.1%) as measured via ISD report, compared with intervention group
*Lisahunter* et al.*, 2014* [38] *Australia*	Quasi-experimental with control group	Students: *n* = 107 Teachers: *n* = 6 Schools: *n* = 1 Classes: *n* = 4	Age approx. 10 years Year 5	Active Kids, Active Minds (AKAM) = additional 30 mins of MPA active break. Dose: once/day	2 terms/approx. 20 weeks	Specially employed PE teacher	Habitual and school day PA: Pedometer (Yamax CW700) School day PA of at least MPA: Accelerometer (ActiGraph)	Cognitive function: Cognitive Assessment System Academic achievement: total score for 8 classroom subjects Classroom behaviour: school behaviour records	Weak	No difference between groups for any of the academic outcomes assessed Physical activity: daily steps declined from pre- (control = 13,772; intervention = 12,447) to post- (control = 12,046; intervention = 9702) for both intervention and control groups
*Whit-Glover* et al.*, 2011* [36] *USA*	RCT	Students *n* = 4599 Schools: *n* = 8	Years 3 to 5	Instant Recess = 10 min MPA active break. Dose: once/day	8 weeks	Teacher	PA during Instant Recess lesson: Direct observation	Classroom behaviour: direct observation	Weak	Classroom behaviour: 11% increase in time spent on-task in intervention, compared with control group Physical activity: MPA increased by 16% and LPA increased by 51%
*Wilson* et al.*, 2015* [37] *Australia*	Within subject	Students: *n* = 58 boys Schools: *n* = 1 Classes: *n* = 4	Mean age: 11.2 years Years 5 & 6	10 min MVPA active break outside the classroom Dose: once/day, 3 times/week	4 weeks	Teacher	PA intensity during active breaks: accelerometer	Sustained attention: 5-min Psychomotor Vigilance Task On-task behaviour: direct observation	Weak	Sustained attention: no difference intervention group pre active break: mean = 477 (285) vs. post active break: mean = 479 (200) Off-task behaviour: no difference: intervention group pre active break: mean = 13.6 (10.0) vs. post active break: mean = 14.8% (11.6)
*Uhrich & Swarm., 2007* [35] *USA*	Quasi-experimental with control group	Students: *n* = 41 Schools *n* = 1 Classes *n* = 2	Age 10 to 11 years Year 5	20 min of sport stacking: using both hands to stack a group of 12 specialized cups in predetermined combinations Dose: 3 times/week	6 weeks	Research staff	None	Decoding and comprehension skills: Gates MacGinitie Reading Test Fourth Edition (GMRT-4) Decoding and Comprehension skill subtests	Weak	Decoding skills: no difference between groups (*F*_1,41_ = 0.03, *p* > 0.05) Comprehension skills: Improvement in intervention group, compared with control (*F*_1,41_ = 4.54, *p* < 0.05)
*Altenburg* et al.*, 2016* [49] *Netherlands*	RCT	Students *n* = 62 Schools *n* = 5 *convenience sample	Aged 10 to 13 years	20 min MPA active breaks comprising video-based dance activities Dose: once per day & twice per day	1 day	Supervised by research staff	PA intensity during active breaks: heart rate monitor	Selective attention: Sky Search sub test of the Test of Everyday Attention for children (TEA-ch test)	Weak	Selective attention: test scores better after 2 bouts (β = −0.26 (95% CI:-0.52,-0.004), compared with one bout (β = 0.06 (95% CI: −0.23,0.36) and control condition. Note: a negative beta indicated a better attention score
*Van den Berg* et al.*, 2016* [50] *Netherlands*	Within subject	Students: *n* = 195 Schools: *n* = 3 Classes: *n* = 8	Age 10 to 13 years Year 5 & 6	12 min MPA active breaks = 3 conditions (aerobic, coordinative & strength-based PA) Dose: once off	3 days	Children followed pre-recorded video of active break sessions, supervised by research staff	PA intensity during active breaks: heart rate monitor	Information processing speed: Letter Digit Substitution Test Selective attention: d2 Test of Attention	Weak	Information processing speed: no change [*F*(1174) = 0.71, *p* = 0.040 Selective attention: no change [*F*(1172) = 0.91, *p* = 0.34
*Mead* et al.*, 2016* [51] *USA*	Quasi-experimental with pre and post testing	Students: *n* = 81 Schools: *n* = 1 Classes: *n* = 3	Year 6 Age 11 to 12 years	3 conditions - implemented during 80 min math class (2 × 5-min active breaks, sitting on stability balls & traditional seated lesson) Dose: every day	Unclear	Teacher	None	Reading, Mathematics and Science: Minnesota Comprehensive Assessments Reading, Mathematics and Language: Measures of Academic Progress	Weak	Reading, Mathematics and Science: no difference between active break (pretest: 527.3 (29.8) vs. posttest (620.9 (34.2) and seated lesson conditions (pretest: 543.9 (13.1) vs. posttest 643.1 (12.4) Reading, Mathematics and Language: no difference between active break (pretest: 219.7 (14.0) vs. posttest (226.8 (15.1) and seated lesson conditions (pretest: 221.2 (16.0) vs. posttest 226.0 (15.1)

Abbreviations

*PA*: physical activity

*LPA*: light intensity physical activity

*MPA*: moderate physical activity intensity

*MVPA*: moderate to vigorous physical activity intensity

*VPA*: vigorous intensity physical activity

*RCT*: randomised controlled trial

**Table 3:** Studies investigating the effect of curriculum focussed active breaks on academic and physical activity outcomes

Paper/ country	Study design	Sample size	Age	Intervention	Duration	Delivery	Physical activity measure	Academic outcome measure	Study quality	Results
*Vazou* et al.*, 2012* [44] *Greece*	Within-subject	*n* = 147	Years 4 to 6	10 mins MPA active break incorporating language arts, math and social studies Dose: unclear	2 weeks	Senior primary education student teachers	None	Academic motivation: Intrinsic Motivation Inventory	Moderate	Academic motivation: Perceived academic competence increased in intervention compared with control. (*F* = 4.87, *p* < 0.05)
*Goh* et al.*, 2016* [57] *USA*	Within-subject	Students: *n* = 210 Classes: *n* = 9 Schools: *n* = 1	Aged 8 to 12 years Year 3 to 5	Take 10! = 10 min active breaks incorporating language arts, math, science, social studies and general health Dose: determined by teacher	8 weeks	Teacher	None	On-task behaviour: direct observation	Moderate	On-task behavior: significant increase in percent on-task behavior from pre-TAKE 10! (82.3 ± 4.5) to post TAKE 10! (89.5 ± 2.7)
*Erwin* et al.*, 2013* [41] *USA*	Quasi-experimental	Students: *n* = 29 Schools: *n* = 1 Classes: *n* = 2	Mean age: 8.87 years Year 3	20 + minutes active break incorporating math and reading Dose: daily	20 weeks	Teacher	Intervention effects of PA on academic outcomes: Pedometer (Walk4Life, LS2500)	Reading and Mathematics: curriculum based measures (CBM), teacher reported grades & standardised test scores (T-PRO, STAR and Discovery Education Assessment)	Moderate	Mathematics: higher scores in intervention compared with control group at time 3 (M_diff_ = 10.87, *p* = 0.003), but not time 1 (M_diff_ = 2.75, *p* = 0.39) or 2 (M_diff_ = 2.16, *p* = 0.49) Reading: higher scores in intervention group at time 1 (M_diff_ = 79.46, *p* < 0.01), time 2 (M_diff_ = 87.41, *p* < 0.01), time 3 (M_diff_ = 92.46, *p* < 0.01) Standardised test scores: Trend towards improvement
*Bailey & DiPerna., 2015* [40] *USA*	Multiple baseline design	Students: Mean of 16 Year 1 and 14 Year 2 students /classroom Schools: *n* = 1 Classes: *n* = 6	Years 1 & 2	Energisers = approx. 10 to 20 min active break incorporating core curriculum Dose: twice daily	5, 7 or 9 weeks	Teacher	School day PA: New-Lifestyles Accelerometer (NL-800):	Intervention acceptability: student and teacher questionnaire	Weak	Intervention acceptability: Most teachers strongly agreed (11%) or agreed (89%) that Energisers did not adversely affect learning. Most teachers strongly agreed (11%) or agreed (67%) that students were better able to pay attention following Energisers 76% of students reported being able to pay better attention in class following Energisers Physical activity: intervention significantly increased school based steps (ES = 0.71 to 1.26)
*Fedewa* et al.*, 2015* [42] *USA*	RCT	Students:> *n* = 460 Schools: *n* = 4	Years 3 to 5	5 min active breaks incorporating core curriculum, totaling 20 mins PA per day Dose: daily	1 year	Teacher	To explain variance in fluid intelligence and academic achievement scores: pedometer (Walk4Life)	Fluid intelligence: Standard Progressive Matrices Reading and Mathematics: Measures of Academic Progress	Weak	Mathematics: improvement in intervention, compared with control group: *t*(33) = 2.17, *p* = 0.04 Reading: improvement in intervention, compared with control group *t*(32) = 1.69, *p* = 0.10 Fluid intelligence: no difference between groups *t*(36) = 0.23, *p* = 0.82
*Grieco* et al.*, 2009* [43] *USA*	Within subject	Students: *n* = 97 Schools: *n* = 1 Classes: *n* = 9	Age 8 to 10 years	Texas I-CAN = one 10–15 min MVPA active break incorporating math, language arts, science, social studies and health Dose: one off lesson	1-day	Teacher	To compare PA among BMI groups: pedometer (Omron HJ 105)	On-task behaviour: direct observation	Weak	On-task behaviour: slight increase after intervention lesson compared with control, although not significant (*F*_1,94_ = 2.19,*p* > 0.10)
*Mahar* et al.*, 2006* [22] *USA*	Cluster RCT with multiple baseline design	Students: *n* = 243 Schools: *n* = 1 Classes: *n* = 15	Kindergarten to Year 4	Energisers = approx. 10 mins active break incorporating core curriculum Dose: daily	4 or 8 weeks	Teacher	School day PA: Pedometer (Yamax SW-200)	On-task behaviour: direct observation (assessed in 2 Year 2, and 2 Year 4 classes)	Weak	On-task behaviour: Improvement in intervention, compared with control group (ES = 0.60). Greatest improvement in on-task behavior for students most off-task (ES = 2.20). Physical activity: intervention group took more in school steps, compared with control group (ES = 0.49)

Abbreviations:

*MVPA*: moderate to vigorous physical activity intensity

*MPA*: moderate physical activity intensity

*PA*: physical activity

*RCT*: randomised controlled trial

**Table 4:** Studies investigating the effect of physically active lessons on academic and physical activity outcomes

Paper/ country	Study design	Sample size	Age	Intervention	Duration	Delivery	PA measure	Academic outcome measure	Study quality	Results
*De Greeff* et al.*, 2016* [32] *Netherlands*	RCT	Students: *n* = 499 Schools: *n* = 12	Years 2 & 3 Mean age: 8.1 ± 0.7 years	Fit & Academically proficient at school = 30 min physically active (MVPA) math and language lessons Dose: 3 x per week	22 weeks per year school, with 1-year and 2-year follow up	1st year - intervention teachers 2nd year –teacher	None	Executive function: Inhibition: Golden Stroop test Working memory: Digit span backward & Visual span backward	Strong	Inhibition: no difference between intervention M = 19.6 (8.1) and control group M = 19.9 (9.5) Digit span backward:no difference between intervention M = 6.0 (2..2) and control group M = 6.2 (1.9) Visual span backward: no difference between intervention M = 6.6 (1.7) and control group M = 6.8 (1.6)
*Riley* et al.*, 2014* [24] *Australia*	Cluster RCT - pilot study	Students: *n* = 54 Classes: *n* = 2 Schools: *n* = 1	Age 10 to 12 years Years 5 & 6	Encouraging Activity to Stimulate Young (EASY) Minds = PA integrated into existing math lessons, 60 mins per lesson Dose: 3 x per week	6 weeks	Research staff	Active lesson and school day PA: Accelerometer (GT3X)	On-task behaviour: direct observation	Strong	On-task behaviour: Greater during intervention lessons, compared with control (19.9% mean difference) Physical activity: 9.7% increase in MVPA across math timeslot, and 8.7% increase across school day
*Riley* et al.*, 2015* [23] *Australia*	Cluster RCT	Students: *n* = 240 Schools: *n* = 8	Age 10 to 12 years Years 5 & 6	EASY Minds = PA integrated into existing math program, 60 mins per lesson Dose: 3 x per week	6 weeks	Teacher	Active lesson and school day PA: Accelerometer (Walk4Life, LS, 2500)	On task behavior: direct observation Mathematics: Progressive Achievement Test	Strong	On-task behaviour: 13.8% increase in intervention compared with control group Mathematics: no difference between groups Physical activity: 2.6% increase in MVPA during math timeslot, and 1.7% increase across school day
*Donnelly* et al.*, 2009* [45] *USA*	Cluster RCT (pre-and post-test)	Students: *n* = 1527 Schools: *n* = 24	Years 2 & 3	Physical Activity Across the Curriculum (PAAC) = MVPA integrated into language, math, science and social studies lessons Dose: 90 min per week, delivered intermittently throughout school day. Approx. 10 mins per session.	3 years	Teacher	School day, weekend day and weekday PA: ActiGraph accelerometer	Academic achievement: subsample (*n* = 454) WIAT-II-A standardised test (math, reading, spelling)	Strong	Academic achievement: improvement in reading, math and spelling scores from baseline to 3 years in intervention, compared with control schools Physical activity: greater school day PA (12%), weekend day PA (17%) and weekday PA (8%) in intervention compared, with control group
*Beck* et al.*, 2016* [33] *Denmark*	Cluster RCT	Students: *n* = 165 Schools: *n* = 3 Classes: *n* = 9	Year 1	Group A = gross motor movements integrated into 60 min math lessons, (e.g. skipping, crawling, throwing while solving math problems) Group B = fine motor movements integrated into 60 min math lessons (e.g. manipulating LEGO bricks while solving math problems) Dose: 3 x per week	6 weeks	Teacher	Physical activity intensity during lessons: Combined heart rate (Polar Team 2 System) and accelerometer (MinimaxX S4) - *Subsample (n = 49)*	Mathematics: standardized test (name not specified)	Moderate	Mathematics: changes in mean math performance were greater for the gross motor group, compared with fine motor group from baseline to intervention end (1.87 ± 0.71). However this affect was not evident from baseline to 8 week follow up.
*McCrady Spitzer* et al.*, 2015* [47] *USA*	Quasi-experimental	Students: *n* = 14 Schools: *n* = 1 Classes: *N* = 1	Age 6 to 7 years Year 1	30–40 min math and language lesson using Active Classroom Equipment Dose: daily	9 months	Teacher	School day PA: Accelerometer	Academic achievement: Dynamic Indicators of Basic Early Literacy Skills (DIBELS)-oral reading fluency, whole words read, correct letter sound	Moderate	Correct letter sound: children in intervention group showed greater improvement (M_diff_ = 45 ± 34) compared with children in the control group (M_diff_ = 15 ± 22) Whole words read: children in intervention group showed greater improvement (M_diff_ = 20 ± 14) compared with children in the control group (M_diff=_7 ± 9) Oral reading fluency: no difference between intervention (M_diff_ = 27 ± 27) and control groups (M_diff_ = 19 ± 16) Physical activity: 46% increase on days used active classroom equipment, compared with days in traditional classroom
*Mullender-Wijnsma* et al.*, 2015a* [49] *Netherlands*	Within subject	Students: *n* = 86 Schools: *n* = 4	Mean age: 8.2 years Years 2 & 3	Fit & Academically proficient at school = 30 min physically active (MVPA) math and language lessons Dose: 3 x per week	22 weeks	Intervention teachers	None	On-task behaviour: direct observation	Moderate	On-task behaviour: higher post intervention, compared with post control lessons (ES = 0.41)
*Graham* et al.*, 2014* [46] *USA*	Non-randomised controlled trial	Students: *n* = 21 Schools: *n* = 1 Classes: *n* = 1	Age 7–8 years Year 2	Jump In! = PA integrated into math lesson Dose: one-off lesson	1 day	Teacher and researcher	None	Mathematics: post session knowledge questionnaire	Weak	Mathematics: no difference between intervention (M = 4.08) and control groups (M = 4.25)
*Mullender-Wijnsma* et al.*, 2015b* [48] *Netherlands*	Quasi-experimental with control group	Students: *n* = 228 Schools: *n* = 6	Mean age: 8.1 years Years 2 & 3	Fit & Academically proficient at school = 30 min physically active (MVPA) math and language lessons Dose: 3 x per week	21 weeks	Intervention teachers	None	Mathematics: speed test arithmetic Reading: 1-min test	Weak	Mathematics: - Year 3: intervention group had higher scores, compared with control group (F[1,99] = 11.72, *p* < 0.05). - Year 2: intervention group had lower scores compared with control group (F[1109] = 12.40, *p* < 0.05) Reading: - Year 3: intervention group had higher scores, compared with control group (F[1,98] = 6.97, *p* < 0.05). - Year 2 no difference between groups (F[1109] = 0.72, *p* = 0.40)
*Norris* et al.*, 2015* [50] *UK*	Quasi-experimental	Students: *n* = 85 Schools: *n* = 2 Classes: *n* = 4	Age 9 to 10 years Year 5	London Olympic theme virtual field trip = 30 mins completing prompted activities (e.g. running 100 m sprint on the spot) Dose: one off lesson	May and June but intervention ran for 1-day in each class	Teacher	Active lesson PA: Accelerometer	Lesson content recall: 10 item content recall quiz	Weak	Content recall quiz: no difference between groups Physical activity: increase in intervention group
*Reed* et al.*, 2010* [51] *USA*	Cluster RCT; pre-and post-test	Students: *n* = 155 Schools: *n* = 1 Classes: *n* = 6	Age 9 to 11 years Year 3	30 mins PA integrated into language and math and social studies lessons. Dose: 3 x per week	3 months	Teacher	DIGI- WALKER pedometer SW 200- used *in intervention group to record steps during lesson only*	Fluid intelligence: Standard Progressive Matrices Academic achievement: Palmetto Achievement Challenge Tests (English, math, science and social studies	Weak	Fluid intelligence: higher scores in intervention, compared with control group (M = 36.66, *p* = 0.45) Social studies: higher scores in intervention, compared with control group (t = *p* = 0.004) Mathematics: no difference between groups (*t* = 1.107, *p* = 0.09) English: no difference between groups (*t* = 0.71, *p* = 0.0478) Science: no difference between groups (*t* = 1.490, *p* = 0.140)
*Grieco* et al.*, 2016* [65] *USA*	Mixed factorial design	Students: *n* = 320 School districts: *n*=1 Classes: *n* = 20	Age 7 to 12 years	Spelling Relay = 10–15 min PA integrated into spelling lessons delivered at different PA intensities (seated traditional lesson, seated game, LMPA game & MVPA game)	1 x lesson per condition	Research staff	Physical activity intensity during lessons: accelerometer	On-task behavior: direct observation	Weak	On-task behaviour: significant increase in time on task from pre- to post- LMPA game (ES = 0.43) and MVPA game (ES = 1.22)
*Mullender-Wijnsma* et al.*, 2016* [66] *Netherlands*	RCT	Students: *n* = 499 Schools: *n* = 12	Years 2 & 3 Mean age: 8.1 ± 0.7 years	Fit & Academically proficient at school = 30 min physically active (MVPA) math and language lessons Dose: 3 x per week	22 weeks per year school, with 1-year and 2-year follow up	1st year - intervention teachers 2nd year –teacher	None	Reading: 1 min test Spelling: spelling scores retrieved from a child academic monitoring system Mathematics: speed test arithmetic and general math scores retrieved from a child academic monitoring system	Weak	Mathematics: intervention group showed greater improvement in math speed test (ES = 0.51) and general math scores (ES = 0.42), compared with control group Spelling: intervention group showed greater improvement in spelling scores (ES = 0.45), compared with control group. Reading: no difference between groups (*t* = 0.00; *p* = 1.00)

Abbreviations:

MVPA: moderate to vigorous physical activity intensity

MPA: moderate physical activity intensity

PA: physical activity

RCT: randomised controlled trial

### Intervention content

There was considerable variation across studies in intervention content. While most (12 out of 19) active break interventions featured basic aerobic movements that students could be performed in their classroom (e.g. jumping jacks), and required no set-up or equipment [24, 35–40, 42, 43, 45, 50, 51], others were performed outside the classroom (e.g. sports field) [26, 41, 46–48], and/or required additional equipment (e.g. markers, skipping ropes, balls, exercise bands, dance videos, or specialised stacking cups) [41, 44, 46, 49]. One study utilised both cognitively engaging active breaks (i.e. physical activity combined with cognitive demand) and active breaks to explore separate and combined effects of physical activity and cognitive engagement on cognitive function [26]. The target frequency, duration and physical activity intensity of the breaks varied, ranging from 4 min of vigorous-intensity physical activity weekly [24, 43] to 20 min of moderate intensity physical activity done twice per day [49].

There was more consistency in content across curriculum-focussed active breaks, compared with the active breaks without curriculum content. All curriculum-focussed active breaks featured physical activity integrated into a combination of key learning areas, including mathematics, language, science and/or social studies, and aimed to reinforce previously taught lesson content [25, 52–57]. Further, most (5 out of 7) required daily participation in 10 to 20 min of physical activity [19, 52–54, 57]. When specified, participation was required at a moderate-[56] or moderate-to vigorous-physical activity intensity [55], but intensity was not specified in the majority (5 out of 7) of these studies [25, 52–54, 57].

While curriculum-focussed active breaks aimed to reinforce previously taught lesson content, physically active lessons were used to teach new lesson content [27, 28, 32, 33, 58–62, 64–66]. These lessons predominately incorporated physical activity into mathematics and/or language lessons, but some also incorporated science and/or social studies [27, 28, 32, 33, 58–62, 64–66]. Lessons ranged in duration from 30 to 60 min [27, 28, 32, 33, 60–64, 66] with most (8 out of 13) requiring participation three times per week [27, 28, 32, 33, 61, 62, 64, 66]. Other physically active lessons were described as single lessons as part of pilot interventions [59, 63, 65], or stipulated physical activity time per week, rather than number of lessons per week [58].

### Intervention fidelity

Intervention fidelity was reported in twelve studies. For the three active break interventions delivered by teachers, various measures of fidelity were used, however, no study clearly reported compliance with implementing active breaks daily or the number of active break sessions conducted. Active break interventions delivered by research staff reported high fidelity, showing most children achieved the required physical activity intensity [39–41], or at least 50% of each intervention session was spent at the required intensity [46, 47].

For physically active lesson interventions, teacher reports showed they delivered lessons either as intended [27] or for at least 50% of the required minutes per week [58]. Similar to active break studies, when delivered by research staff, at least 60% of intervention lessons were spent at the required physical activity intensity [61, 62]. No curriculum focussed active break study reported fidelity.

### Methodological quality

Of the 39 identified studies, most (36 out of 39) received a moderate [24, 26, 33, 38–41, 48, 53, 55–57, 60, 62], or weak quality rating score [25, 27, 28, 35–37, 42, 44–47, 49–52, 54, 59, 61, 63–66]. Three received a strong quality rating score [32, 43, 58]. Low to moderate quality score ratings were mostly attributable to not reporting or controlling for relevant demographic confounders, not reporting blinding of participants and researchers, and not reporting participant attrition. Further, for many studies, authors did not report the rate of participant or school participation. See Appendix A for further detail on quality assessment of included studies.

### Academic-related outcomes: Classroom behaviour

**Fig. 2:**
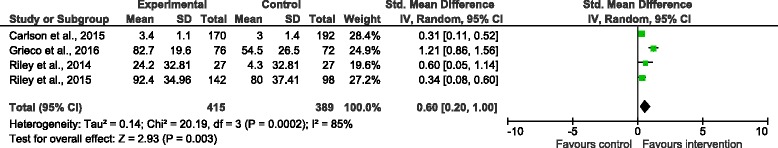
Forrest plot of the effect of classroom-based physical activity on classroom behaviour

Studies assessed the effect of participation in these programs on academic-related outcomes both immediately following participation in a session (acute) and after a longer exposure (chronic; e.g. pre- and post- intervention periods spanning up to 8 months). Regardless of type of classroom-based physical activity, the majority of studies (10 out of 12) showed participation in these programs had an acute effect on improving on-task classroom behaviour [25, 27, 28, 39, 52, 57, 62, 65] and reducing off-task behaviour [36, 43] However, evidence in the few studies with longer term follow-up (2 out of 2 studies) suggest that this improvement may dissipate over time, with no difference between groups when chronic intervention effects on reported behaviour incidents were assessed [42, 47]. Due to few studies investigating chronic effects of classroom-based physical activity on on-task and off task classroom behaviour (<5) it was not possible to separate acute and chronic effects in the meta-analysis. Results from the 4 included studies show classroom-based physical activity had a positive effect on improving on-task behaviour and reducing off-task behaviour (standardised mean difference = 0.60 (95% CI: 0.20,1.00)) (see Fig. 2).

### Academic-related outcomes: Cognitive function

**Fig. 3:**
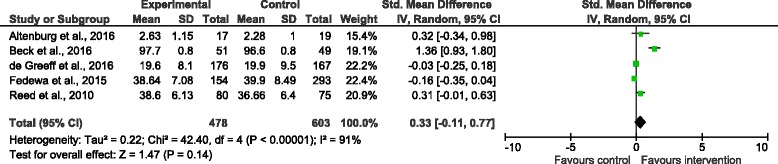
Forrest plot of the effect of classroom-based physical activity on cognitive function

Studies also assessed acute and chronic effects of classroom-based physical activity on a range of cognitive functions [24, 32, 37, 38, 40, 41, 46, 47, 49, 50, 54, 64]. Results showed active breaks had an acute positive effect on selective attention (3 out of 4 studies) [24, 41, 49]. No acute effect was reported for sustained attention [46], information processing [50] or focussed attention, processing speed and accuracy [26], and no chronic effect was reported for planning, attention, simultaneous or successive cognitive processes [47] or executive function [32]. Acute intervention effects on executive function were inconsistent, with no difference between groups reported in one study [40], while another reported improvements in executive function but only for those receiving the intervention in the second week of delivery [37, 38]. Results were also inconsistent for chronic intervention effects on fluid intelligence, with one study reporting a significant improvement after 3 months [64], while another reported no difference between groups after 1-year [54]. Due to few studies reporting chronic effects of participation (<5) results for acute and chronic studies were combined in the meta-analysis (5 studies). Results from the meta-analysis indicate classroom-based physical activity had no effect on cognitive function (standardised mean difference = 0.33 (95% CI: -0.11,0.77) (see Fig. 3).

### Academic-related outcomes: Academic achievement

**Fig. 4:**
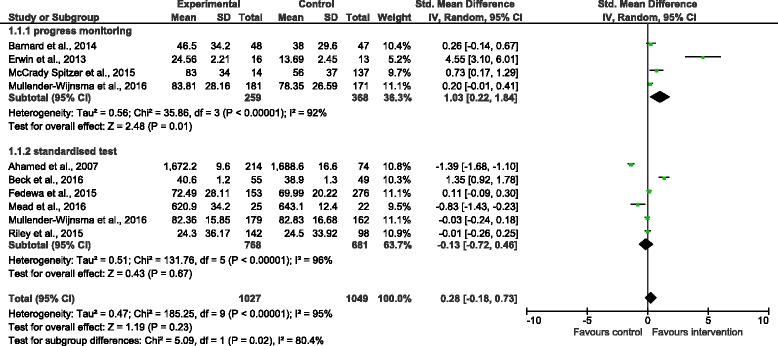
Forrest plot of the effect of classroom-based physical activity on academic achievement

Studies assessed intervention effects on academic achievement using a range of academic assessment tools, including standardised tests, progress monitoring tools, grades and content recall quizzes. Reported effects on academic achievement varied by intervention duration and the type of assessment tool used. Interventions of shorter duration tended to show improvement in academic achievement if a progress monitoring tool was used, but not if a national standardised test was used. Seven out of 8 studies using a progress monitoring tool reported significant improvement in academic achievement following intervention periods ranging from 4 weeks to 1-year [40, 44, 53, 54, 60, 61, 66]. In contrast, most (4 out of 7) studies indicated no difference between groups following intervention periods less than 1-year when national standardised tests were used as the outcome measure [27, 42, 64, 66]. However, standardised test scores significantly improved following a 1-year [51] and 3-year physically active lesson intervention [58]. These results were confirmed in the meta-analysis. When progress monitoring tools were used (4 studies) as the outcome measure, academic-related outcomes generally showed improvement (standardised mean difference = 1.03 (95% CI: -0.22,1.84)). However, when measured using a national standardised test (6 studies), academic-related outcomes generally showed no improvement (standardised mean difference = −1.13 (95% CI: -0.72,0.46)) (see Fig. 4).

In addition to standardised tests and progress monitoring tools, a small number of studies (not included in the meta-analysis) measured academic achievement via grades, content recall quizzes and self-reported academic competence. Results were inconsistent. One study reported no difference between groups for grades across eight subjects (total score) following a 20-week active break program [47], Another reported a greater proportion of students in the control group showed improvement in grades for math and reading, compared with an active break intervention group [42]. Other studies assessed academic achievement via content recall quizzes and perceptions of academic competence, with no difference between groups in math and social studies scores following participation in single lessons lasting between 10 and 30 min [59, 63]. Another study reported self-reported perceptions of academic competence improved during physically active lessons [56].

### Dose response relationship

Four studies aimed to explore the optimal dose of active break (i.e. amount of physical activity required to confer academic benefits) required to provide maximum effects on academic-related outcomes, by manipulating intensity [41], duration [39, 40], and frequency [49] of active break sessions. Howie and colleagues [39, 40] compared 5-, 10- and 20-min active breaks with a 10-min no break condition. Results showed on-task classroom behaviour significantly improved after the 10-min active break condition [39] and math scores were highest after the 10-min (ES = 0.24) and 20-min (ES = 0.27) active break conditions [40]. Janssen et al. [41] compared selective attention scores across 15 min of each of the following four conditions: no break (continued with school work), passive break (teacher read story), moderate-intensity active break (jogging, passing, dribbling), and vigorous-intensity active break (running, jumping, skipping) [41]. Results showed that selective attention scores improved most after the moderate-intensity active break [41]. Altenburg and colleagues [49] compared acute effects of different frequencies (one per day vs. twice per day) of 20 min moderate-intensity active breaks. Results showed significantly better selective attention scores for children who received the twice per day frequency [49].

### Physical activity outcomes

**Fig. 5:**
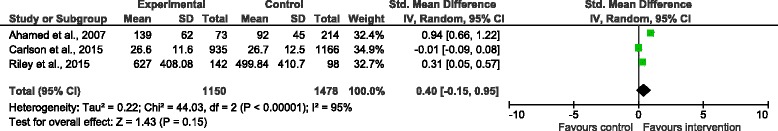
Forrest plot of the effect of classroom-based physical activity on physical activity

Eleven studies examined the effect of classroom-based physical activity interventions on children’s physical activity levels using a range of measures, including questionnaire [35], direct observation [45], pedometer [25, 47, 52], and accelerometer [27, 28, 36, 47, 58, 60, 63]. Across most (10 out of 11) classroom-based physical activity interventions, small increases in physical activity were reported [25, 27, 28, 35, 36, 45, 52, 58, 60, 63]. Across studies there was a 2% to 16% increase in moderate- to vigorous- intensity physical activity during intervention lessons, [27, 28, 45, 60, 63], and 2% to 12% increase in school day moderate- to vigorous- intensity physical activity [27, 28, 58]. However, as shown in Fig. 5 results from 3 studies included in meta-analysis indicate classroom-based physical activity did not affect physical activity (standardised mean difference = 0.40 (95% CI: -0.15,0.95).

## Discussion

A systematic search of the literature found 39 studies assessing the effect of classroom-based physical activity on academic-related outcomes, including classroom behaviour, cognitive function and academic achievement. In the majority of studies, academic-related outcomes improved following participation in classroom-based physical activity programs. These findings are generally consistent with earlier reviews finding that overall physical activity level was either positively associated, or was not associated with academic-related outcomes [14, 15, 17]. In addition, the interventions included in the current review generally resulted in more physical activity.

The finding that classroom-based physical activity improves on-task or reduces off-task classroom behaviour immediately following participation in intervention sessions is consistent with previous reviews of school-based physical activity. For example, systematic reviews of the effect of physical activity during the school break time on academic-related outcomes showed positive associations between participation in physical activity before class (e.g. during recess/snack time) and on-task classroom behaviour in subsequent lessons [17, 29]. Therefore, breaking up lesson time with physical activity offers a promising strategy to improve on-task behaviour. Further, physically active lessons may provide a strategy to engage students in lesson content, which may lead to improved on-task classroom behaviour. However, this assumption is purely speculative and further research is needed to confirm this. One study reported a non-significant increase in on-task classroom behaviour after intervention sessions, compared with control [55]. A possible reason for this finding may be that the sample size in that study (*n* = 97) may not have been large enough to detect a significant improvement. Few studies (*n* = 3) reported that classroom-based physical activity had no effect on classroom behaviour. The majority of these studies (2 out of 3) reported that, while behaviour incidents and off-task behaviour increased in both the intervention and control groups, the increase was greater in the control group, compared with the intervention group [46, 47]. These findings may encourage teachers to consider implementing classroom-based physical activity programs by alleviating concerns about reducing on-task behaviour due to the disruption to the classroom routine [10].

While classroom-based physical activity showed relatively consistent positive associations with classroom behaviour, effects on cognitive function were inconsistent. A possible explanation for this finding may relate to the variability in the quality of measures used. Overall results showed studies that reported improvements in cognitive function used measures with moderate to high levels of reliability and validity [67, 68]. In contrast, studies reporting no improvement in cognitive function mainly used measures with lower levels of reliability and validity [69–71]. It may be important for future studies to use tests of cognitive function with established validity and reliability.

A further possible explanation for inconsistent effects on cognitive function may relate to the level of cognitive engagement inherent in each type of classroom-based physical activity. It has been suggested that cognitively engaging physical activity (i.e. physical activity combined with cognitive demands) may enhance cognitive function to a greater degree than non-cognitively engaging physical activity (e.g. repetitive exercise) [72]. As curriculum-focused active breaks and physically active lessons can be considered cognitively engaging physical activity, it could be hypothesised that these types of classroom-based physical activity would lead to greater improvements in cognitive function, compared with active breaks that involve no cognitive content. While the majority of physically active lesson and curriculum focussed active break interventions (2 out of 3 studies) and only half of active break interventions (5 out of 10 studies) led to improvements in cognitive function, there were too few cognitively engaging interventions included in the review to draw a definitive conclusion. The one study that compared cognitively engaging and non-cognitively engaging active breaks, showed an impact on cognitive outcomes for the cognitively engaging breaks group only, lending support to this hypothesis [26]. Although not explicitly stated, many studies which do not purport to involve cognitively engaging physical activity involve some activities which are likely to confer cognitive engagement e.g. hopping sequences to music [37, 38], and coordinative exercises [50]. Some of these report positive and some null findings, yet it is difficult to ascertain the proportion of physical activity children were exposed to that was cognitively engaging. Future studies are encouraged to separate the effects of cognitively engaging and non-cognitively engaging physical activity on cognitive functions.

In addition to the cognitive test used, results may be dependent on the type of cognitive function assessed. For example, classroom-based physical activity appeared to have a particularly beneficial effect on selective attention [24, 41, 49], compared with other components of cognitive function, including sustained attention [46], fluid intelligence [54, 64], information processing speed [50], and executive function [32, 37, 38, 40]. However, a recent systematic review concluded that there is insufficient evidence to conclude what specific cognitive functions are most affected by physical activity [73]. Exercise-induced arousal may provide a further explanation for inconsistency in findings. This theory suggests that the heightened level of arousal during physical activity facilitates cognitive function and that this effect may be moderated by physical activity intensity [74]. However, while the majority of included studies reported a target physical activity intensity, few measured physical activity intensity during interventions precluding conclusions regarding the role of physical activity intensity on cognitive function. Thus, the favourable effect of physical activity on selective attention indicated in this review requires further research for confirmation. Nonetheless, should improvements in selective attention occur, such as the ability to ignore distractions this may be of particular interest to teachers and may provide motivation to incorporate physical activity into their classroom routine.

In addition to classroom behaviour and cognitive function, classroom-based physical activity may also have a positive effect on academic achievement. However, effects on academic achievement may be dependent on intervention duration and the type of assessment tool used to measure academic achievement. In the current review it appeared that interventions of shorter duration were more likely to show an improvement in academic achievement if a progress monitoring tool was used, rather than a national standardised test. This may be because curriculum-based measures are sensitive to small changes in academic achievement, and can be administered frequently (e.g. weekly) [75, 76], while standardised tests are usually designed to be administered less frequently (e.g. yearly), and are not sensitive to short-term progress. Therefore, progress monitoring tools may be a more suitable choice to determine intervention effects on academic achievement in the short-term. This finding has important implications for future research, indicating it may be important to consider intervention duration when selecting the measure of academic achievement. Therefore, future intervention studies may consider using a progress monitoring tool for intervention periods less than 1-year, and standardised tests for intervention periods longer than 1-year if academic achievement is the outcome of interest.

Other studies investigated the impact of different doses of classroom-based physical activity on academic-related outcomes. However, results are based on few (*n* = 4) heterogeneous studies which considered a limited range of potential physical activity doses. Thus, further research is needed to be able to draw conclusions regarding the minimal dose of active break required to impact academic-related outcomes.

Several studies aimed to explore the effect of classroom-based physical activity on children’s physical activity levels [25, 27, 28, 35, 36, 45, 47, 52, 58, 60, 63]. Results from the meta-analysis showed classroom-based physical activity did not affect physical activity levels. However, as only three of the 11 identified studies could be included in the meta-analysis these results should be interpreted with caution, and further research is warranted. Findings from the systematic review consistently revealed small increases in physical activity in children participating in the intervention, compared with students in the comparison group. These findings are in line with results from another review reporting positive associations between classroom-based physical activity interventions and children’s physical activity levels [21]. While promising, it is possible compensation for this activity occurs outside of school. However, with limited information available, it is difficult to make strong conclusions on this. Further, it can be difficult to implement physical activity interventions in schools, often due to a lack of time associated with competing curriculum demands [77]. However, classroom-based physical activity is unique from other forms of school-based physical activity (e.g. Physical Education class and school sport) in that it does not compete for instructional time (physically active lessons and curriculum-focussed active breaks) or requires only minimal time commitment (active breaks). Thus, classroom-based physical activity may be a potentially appealing option for schools as it offers a time-efficient strategy to promote physical activity.

## Limitations

The considerable variation between studies in study designs, intervention content and outcome assessment tools make it difficult to draw definitive conclusions, as evidenced by the small proportion of studies that could be included in meta-analyses. For studies that assessed intervention effects on physical activity, the majority compared physical activity levels during the classroom-based physical activity session, with a traditional seated lesson [27, 28, 45, 47], or assessed intervention effects on school day physical activity levels only [25, 27, 28, 36, 52, 60]. Therefore, it is unclear if the increase in physical activity during these sessions is compensated for by a reduction in physical activity at other times of the day. However, as intervention effects on improving on-task, reducing off-task classroom behaviour and cognitive function appear to be primarily acute, this may not be a problem for these outcomes. In addition, few studies used an objective measure of physical activity intensity [27, 28, 35, 36, 47, 58, 60, 63]. Thus, future studies using objective measures of physical activity are required to determine intervention effects on overall moderate- to- vigorous-intensity physical activity, and to determine intervention fidelity (i.e. if the required physical activity intensity is met) within the sessions. Lastly, given that the majority of included studies reported significant improvements in academic-related outcomes, it is possible publication bias may have impacted the lack of published null associations.

## Conclusion

Classroom-based physical activity interventions may provide a practical, low-cost, and effective strategy to increase academic-related outcomes, particularly acute positive effects on improving on-task and reducing off-task classroom behaviour and selective attention. Classroom-based physical activity could also have the potential to increase children’s physical activity levels, however further research is needed to confirm this. Findings from this systematic review should be interpreted with caution given the high number of included studies of low methodological quality, suggesting there is room for improvement in classroom-based physical activity intervention study designs and reporting. This review has identified a number of areas for further research in order to increase understanding of the effect of classroom-based physical activity on academic and physical activity outcomes. These include the need for future studies to use objective measures of physical activity, and to consider intervention duration when selecting a measure of academic achievement. In addition, future studies should explore the effect of classroom-based physical activity interventions on specific cognitive outcomes, as well as the impact of different types of physical activity (aerobic versus anaerobic versus resistance training and cognitively engaging vs. non-cognitively engaging physical activity) on academic-related outcomes. Further, it is not clear if improvements in academic-related outcomes are a result of the physical activity or a result of the break from academic instruction, therefore future research is encouraged to add an attention control group. Lastly, it is recommended future studies use a standardized measure of cognitive function with established reliability and validity to be able to make comparisons across studies.

## Appendix

**Table 5:** Quality assessment of included studies

Paper	Selection bias	Study design	Confounders	Blinding	Data collection methods	Withdrawals & dropouts	Overall
*Goh* et al.*, 2016*	moderate	moderate	strong	moderate	moderate	weak	MODERATE
*Beck* et al.*, 2016*	moderate	strong	strong	moderate	weak	strong	MODERATE
*De Greeff* et al.*, 2016*	moderate	strong	strong	moderate	strong	moderate	STRONG
*Altenburg* et al.*, 2016*	weak	strong	strong	moderate	strong	weak	WEAK
*Mead* et al.*, 2016*	weak	strong	weak	moderate	weak	weak	WEAK
*Mullender Wijnsma* et al.*, 2016*	moderate	strong	weak	weak	strong	strong	WEAK
*Van den berg* et al.*, 2016*	weak	strong	strong	moderate	moderate	weak	WEAK
*Grieco* et al.*, 2016*	moderate	strong	weak	strong	moderate	weak	WEAK
*Carlson* et al.*, 2015*	moderate	weak	weak	weak	strong	strong	WEAK
*Ma* et al.*, 2015*	moderate	moderate	strong	moderate	strong	weak	MODERATE
*Ma* et al.*, 2014*	moderate	moderate	strong	moderate	moderate	strong	STRONG
*Howie* et al.*, 2014*	moderate	moderate	strong	moderate	strong	weak	MODERATE
*Howie* et al.*, 2015*	moderate	moderate	strong	moderate	strong	weak	MODERATE
*Janssen* et al.*, 2014*	weak	moderate	strong	moderate	strong	weak	MODERATE
*Wilson* et al.*, 2015*	moderate	moderate	strong	moderate	weak	weak	WEAK
*Hill* et al.*, 2011*	moderate	moderate	strong	strong	weak	strong	MODERATE
*Hill* et al.*, 2010*	moderate	moderate	strong	strong	weak	weak	WEAK
*Ahamed* et al.*, 2007*	moderate	strong	strong	moderate	weak	weak	WEAK
*Whitt-Glover* et al.*, 2011*	moderate	strong	weak	moderate	weak	strong	WEAK
*Uhrich & Swarm., 2007*	moderate	strong	weak	moderate	strong	weak	WEAK
*Katz* et al.*, 2010*	moderate	strong	weak	moderate	weak	weak	WEAK
*Lisahunter* et al.*, 2014*	weak	strong	weak	moderate	strong	weak	WEAK
*Bernard* et al.*, 2014*	moderate	strong	weak	moderate	strong	strong	MODERATE
*Fedewa* et al.*, 2015*	weak	strong	weak	moderate	strong	strong	WEAK
*Erwin* et al.*, 2013*	moderate	strong	weak	moderate	strong	strong	MODERATE
*Grieco* et al.*, 2009*	moderate	moderate	strong	moderate	moderate	weak	MODERATE
*Mahar* et al.*, 2006*	moderate	strong	weak	moderate	moderate	weak	WEAK
*Bailey & DiPerna., 2015*	moderate	moderate	strong	moderate	weak	weak	WEAK
*Vazou* et al.*, 2012*	moderate	moderate	strong	moderate	strong	weak	MODERATE
*McCrady-Spitzer* et al.*, 2015*	weak	moderate	strong	moderate	strong	strong	MODERATE
*Norris* et al.*, 2015*	moderate	strong	strong	moderate	weak	weak	WEAK
*Mullender Wijnsma* et al.*, 2015a*	moderate	moderate	strong	moderate	moderate	weak	MODERATE
*Mullender Wijnsma* et al.*, 2015b*	moderate	strong	weak	moderate	moderate	weak	WEAK
*Graham* et al.*, 2014*	weak	strong and moderate	weak	moderate	weak	weak	WEAK
*Riley* et al.*, 2014*	moderate	strong	weak	weak	weak	strong	WEAK
*Riley* et al.*, 2015*	moderate	strong	strong	weak	weak	strong	WEAK
*Donnelly* et al.*, 2009*	moderate	strong	strong	moderate	strong	strong	STRONG
*Reed* et al.*, 2010*	weak	strong	weak	moderate	strong	weak	WEAK
*Schmidt* et al.*, 2016*	moderate	strong	strong	moderate	strong	weak	MODERATE

Overall rating

Strong = no weak ratings

Moderate = 1 weak rating

Weak = 2 or more weak ratings
